# Nebulized enriched heparin to treat no critical patients with Sars-Cov-2: Triple-blind clinical trial

**DOI:** 10.1097/MD.0000000000028288

**Published:** 2021-12-23

**Authors:** Matheus Bertanha, Lenize da Silva Rodrigues, Pedro Luciano Mellucci Filho, Andrei Moroz, Maria Inês de Moura Campos Pardini, Marcone Lima Sobreira, Edison Luiz Durigon, Rafael Rahal Guaragna Machado, Rejane Maria Tommasini Grotto, Marcelo Andrade de Lima, Helena Bonciani Nader, Marli Leite de Moraes, Alexandre Naime Barbosa, Natália Bronzatto Medolago, Fábio Florença Cardoso, Angelo José Magro, Cristiane Rodrigues Guzzo Carvalho, Leonardo Nazário de Moraes, Rita de Cássia Alvarado, Helga Caputo Nunes, Gustavo Constantino de Campos, Vinicius Tadeu Ramos da Silva Grillo, Nathalia Dias Sertorio, Carlos Magno Castelo Branco Fortaleza

**Affiliations:** a Department of Surgery and Orthopedics, São Paulo State University – UNESP, Botucatu Medical School, Botucatu, SP, Brazil; b Applied Biotechnology Laboratory, Research Nucleus of Clinical Hospital, São Paulo State University – UNESP, Botucatu Medical School, Botucatu, SP, Brazil; c Department of Bioprocess and Biotechnology, São Paulo State University – UNESP, School of Pharmaceutical Sciences, Araraquara, SP, Brazil; d Internal Medicine Division, São Paulo State University – UNESP, Botucatu Medical School, Botucatu, SP, Brazil; e Department of Microbiology, Institute of Biomedical Sciences, University of São Paulo – USP, São Paulo, SP, Brazil; f Scientific Platform Pasteur, University of São Paulo – USP, São Paulo, SP, Brazil; g Bioprocessing and Biotechnology Department, São Paulo State University – UNESP, School of Agriculture, Botucatu, SP, Brazil; h Molecular & Structural Biosciences, School of Life Sciences, Keele University, Newcastle-Under-Lyme, Staffordshire, UK; i Department of Biochemistry, Federal University of São Paulo – UNIFESP, São Paulo, SP, Brazil; j Institute of Science and Technology, Federal University of São Paulo – UNIFESP, São José dos Campos, SP, Brazil; k Department of Infectious Diseases, São Paulo State University – UNESP, Botucatu Medical School, Botucatu, SP, Brazil; l Clinical Research Unit, São Paulo State University – UNESP, Botucatu Medical School, Botucatu, SP, Brazil; m Biosciences Institute, São Paulo State University – UNESP, Botucatu, SP, Brazil; n Quality control laboratory, Cellavita Scientific Research, Valinhos, SP, Brazil; o Department of Orthopedics and Traumatology, University of Campinas – UNICAMP, School of Medical Sciences, Campinas, SP, Brazil.

**Keywords:** antiviral agents, clinical protocol, COVID-19, heparin, inhalation, SARS-CoV-2

## Abstract

**Background:**

Coronavirus disease 2019 (COVID-19) is a viral respiratory disease that spreads rapidly, reaching pandemic status, causing the collapse of numerous health systems, and a strong economic and social impact. The treatment so far has not been well established and there are several clinical trials testing known drugs that have antiviral activity, due to the urgency that the global situation imposes. Drugs with specific mechanisms of action can take years to be discovered, while vaccines may also take a long time to be widely distributed while new virus variants emerge. Thus, drug repositioning has been shown to be a good strategy for defining new therapeutic approaches. Studies of the effect of enriched heparin in the replication of severe acute respiratory syndrome Coronavirus 2 (SARS-CoV-2) in vitro assays justify the advance for clinical tests.

**Methods and analysis:**

A phase I/II triple-blind parallel clinical trial will be conducted. Fifty participants with radiological diagnosis of grade IIA pneumonia will be selected, which will be allocated in 2 arms. Participants allocated in Group 1 (placebo) will receive nebulized 0.9% saline. Participants allocated in Group 2 (intervention) will receive nebulized enriched heparin (2.5 mg/mL 0.9% saline). Both groups will receive the respective solutions on a 4/4 hour basis, for 7 days. The main outcomes of interest will be safety (absence of serious adverse events) and efficacy (measured by the viral load).

Protocols will be filled on a daily basis, ranging from day 0 (diagnosis) until day 8.

## Abstract 2

Supplemental Digital Content is available in the text

## 1 Introduction

The first cases of coronavirus in humans were initially identified in 1937 and their particular crown-like morphology was demonstrated in 1965.^[1]^ A new coronavirus, named as severe acute respiratory syndrome coronavirus 2 (SARS-CoV-2), was discovered in December 2019 at Wuhan province, China, is the causative agent of the coronavirus disease 19 (COVID-19).^[2]^ The disease spreads rapidly worldwide, reaching a pandemic status, causing a significant amount of occurrences related to severe respiratory failure, which can quickly progress to the need for mechanical ventilation assistance.^[3,4]^ The severity of the cases and the need for highly complex medical and hospital resources, associated with a large number of cases due to the high infectivity of the virus, has led numerous health systems to their maximum support capacity. This caused the collapse of health services and strong socioeconomic impact worldwide related to health policies and social isolation measures imposed to decrease the accelerated progression of the disease.

### 1.1 Rationale

Heparin is naturally polydisperse and heterogeneous acidic polysaccharide member of the glycosaminoglycans family.^[5]^ It has been used as an anticoagulant for over 80 years, with well-established safety, stability, bioavailability, and pharmacokinetics. Recent studies have shown the impact of pharmacological thromboprophylaxis and systemic anticoagulation as effective in protecting thrombotic events in critically and non-critically COVID-19 patients.^[6–8]^ In addition to its anticoagulant nature, it also has other clinical applications that are not completely explored, including antiviral activity against flavivirus,^[9,10]^ herpes,^[11]^ influenza^[12]^ and HIV,^[13,14]^ and, more recently, evidenced antiviral action against SARS-CoV.^[15]^

The interactions that occur between viral surface proteins and host cell receptors are the basis for viral infectivity and transmissibility.^[16]^ SARS-CoV-2 expresses the surface glycoprotein S1 (spike protein), which contains a receptor binding domain (RBD) that interacts with the angiotensin-converting enzyme 2 (ACE2), enabling the onset of infection.^[17]^ Previous studies demonstrated that heparin and its analogues are able to bind to the SARS-CoV-2 spike protein and block SARS-CoV-2 infection using therapeutic relevant concentrations. Such inhibition is likely to be caused by overlapping heparin/heparan sulfate (HS) binding sites on the subunit S1 of the spike protein, where the RBD is located, which restrict S1 binding to the ACE2 receptor.^[18]^

Pharmaceutical unfractionated heparin formulations contain polysaccharide chains of molecular weight ranging from ∼5000 to ∼30,000 Da.^[19]^ We have developed a method to enrich commercially available unfractionated heparin by simple filtration, where heparin formulations are filtered and cleared by centrifugation (Amicon Ultra-15 Centrifugal filters 10kDa, Merck Millipore, Burlington, Massachusetts, United States of America) to obtain an enriched heparin preparation, produced in a clean area and sterilized by filtration with a 0.22 filter. The enriched heparin shows an antiviral activity in vitro (data not shown) (standardized VERO cell-based assays with a clinical isolate SARS-CoV-2/SP02/human/2020/BRA (GenBank access no. MT126808.1)). Upon heparin treatment, there was a significant decrease in viral load (≥75%) with no observed cytotoxicity for the tested doses (62.5–250 μg/mL). On the basis of the findings of this in vitro evaluation, we conceived the hypothesis that this enriched heparin preparation could be effective to inhibit SARS-CoV-2 viral replication in infected patients, superior to the inhibition by conventional unfractionated heparin at low doses.

### 1.2 Inhaled heparin

The mechanism of nebulized heparin has been tested in several clinical studies in the last 2 decades, mainly for the treatment of acute airway inflammation caused by smoke inhalation and severe acute respiratory diseases.^[20]^ Due to its systemic anticoagulant nature, the administration of heparin by inhalation presented an initial concern regarding the risk of bleeding during treatment. However, it is known that the systemic absorption of heparin by this route is minimal^[21]^.

In the study carried out by Bendstrup et al,^[21]^ the inhibitory effects over systemic coagulation by nebulized unfractionated heparin at doses up to 64 mg were evaluated. This study showed that there is a dose-dependent effect on factor Xa inactivation with minimal changes in the activated partial thromboplastin time (aPTT) and indicated the safety of unfractionated heparin nebulization for clinical or research purposes, without increasing the risk of bleeding.^[22]^ Phelps et al^[23]^ recently carried out a systematic review about the use of unfractionated heparin inhalation for the treatment of heat injuries in the respiratory system of burn patients and concluded that nebulized heparin did not demonstrate an increase in the risk of bleeding rates and decreased the duration of mechanical ventilation.

For COVID-19, the heparin anti-viral effect is expected to be related to the fact that heparin blocks viral interaction with host cell receptors thereby inhibiting viral interaction with the cell and, consequently, the viral infection.^[24,25]^ Furthermore, there are other important mechanisms that are relevant for the potential effects of inhaled heparin in COVID-19, including effects on inflammation, on coagulation, on reduction of DNA NETS, and on mucus.^[26]^ To this moment, some studies indicate that the treatment with systemic heparin reduces hospital mortality in patients with COVID-19^[27]^ and strongly support the clinical investigation of heparin as a potential treatment for patients with COVID-19.^[28,29]^ Thus, these findings justify further investigation to deepen the knowledge about the clinical effects of different heparin preparations.

### 1.3 General objectives

The main objective of this study is to evaluate the safety and efficacy of the use of nebulized enriched heparin administered in patients with SARS-CoV-2.

### 1.4 Specific objectives

The specific objectives of this study are as follows:

1. To assess the anticoagulation profile of treated patients;
2. To assess the viral load of the SARS-CoV-2 virus of treated patients;
3. To assess the inflammatory profile of the treated patients;
4. To assess the infectious profile of the treated patients;
5. To evaluate the respiratory evolution of treated patients;
6. To assess the clinical evolution of treated patients, including the need for an intensive care unit (ICU).

### 1.5 EnHanCed study design

The present protocol refers to a phase I/II, prospective, randomized, parallel, triple-blind clinical trial. This design is justified by the lack of a drug-specific, cost-efficient treatment for SARS-CoV-2 patients and is expected to evaluate safety of the intervention. The use of a therapeutic drug justifies the need for a placebo control group. Triple-blind clinical studies have the lowest levels of bias and, therefore, the highest level of evidence for a clinical study. It is understood that there is no need for a purely phase I study based on the literature data previously presented.

## 2 Methods and analysis

### 2.1 Study setting

The study will be carried out at the São Paulo State University (UNESP) Clinical Hospital of the Botucatu Medical School, Botucatu, São Paulo, Brazil.

### 2.2 Eligibility criteria

Eligibility criteria are as follows:

1. Sign and agree to the free and informed consent form;
2. Both genders, of any ethnic origin, aged between 18 and 90 years;
3. COVID-19 infected, diagnosed by reverse-transcriptase polymerase chain reaction (RT-PCR) and/or with a strong suspicion of COVID-19, according to compatible clinical and radiological findings;
4. Time of disease evolution less than 14 days;
5. Radiological diagnosis of grade IIA pneumonia, with gas exchange ratio >200 in blood gas analysis (paO_2_ /pFiO_2_), characterizing mild hypoxemia;
6. Indication of hospital treatment regime, provided that the period of hospitalization before inclusion is not more than 48 hours;
7. Demand for supplemental oxygen therapy (O_2_) less than 5 L/min.

### 2.3 Exclusion criteria

Exclusion criteria are as follows:

1. Disagree with the terms of the study;
2. Moderate or severe respiratory failure requiring ICU admission requiring invasive mechanical ventilation or positive pressure noninvasive ventilation (NIV);
3. Pregnancy or puerperium;
4. Hematological diseases;
5. Coagulation disorders;
6. Previous use of anticoagulants in therapeutic dosages;
7. Previous heparin-induced allergy or thrombocytopenia or current thrombocytopenia with a count of less than 50,000 platelets/mm^3^;
8. COVID-19 vaccinated patients.

### 2.4 Interventions

Considering the pulmonary water loss of 8 to 10 mL during the gas exchange in 15 minutes (of inhalation) and compensating the dilution of the solution in the pulmonary fluids, we opted for the dose of 2.5 mg heparin in 1 mL of 0.9% saline, aiming the effective mean tested doses (62.5–250 μg/mL), jet nebulized (atomizers) 4/4 hours, except the nebulization that would be administered between 12 pm and 6 am. The treatment will be done with 5 mL of this solution previously prepared (2.5 mg heparin in 1 mL of 0.9% saline - totalizing 12.5 mg of enriched heparin per cycle), for 7 days consecutively, We assumed that any reductions in viral load promoted by enriched heparin would have a significant impact during the first 14 days of the infectious process. We considered that most patients have developed the disease for at least 7 days prior to being admitted and that the viral load tends to reduce spontaneously after 14 days. For those reasons, and looking to mitigate any prolongation of hospital stay that we could cause, we opted for a period of 7 days of treatment. All measures for containment of aerosol dispersion during nebulization including unidirectional inspiration and expiration valves.

Individual sterile vials containing 5 mL of enriched heparin (2.5 mg/mL, which will be adjusted after the filtration process based on the result of biochemical analysis) or placebo (adding to the same total volume of 0.9% saline) will be prepared by the pharmacist, stored in a freezer at -30°C, and delivered to the research team in a blind character. After receiving the preparation (enriched heparin or placebo), these will be inserted into the nebulizer cup and administered in a source of oxygen (5 L/min or according to the patient's need) in isolated rooms, a process that takes 15 minutes. Nebulization therapy will be performed after isolation of the patient and with support from the hospital infection control committee, so that there will be no greater risk of spreading the virus.

1. Group 1 - Control: Jet nebulization with 5 mL of 0.9% saline, administered every 4 hours for 7 days, except the nebulization that would be administered between 12 pm and 6 am (5 inhalations per day).
2. Group 2 - Experimental: Jet nebulization with 5 mL of enriched heparin (2.5 mg/mL diluted in 0.9% saline – totalizing 12.5 mg of enriched heparin per cycle), administered every 4 hours, for 7 days, except the nebulization that would be administered between 12 pm and 6 am (5 inhalations per day).

Between 12 pm and 6 am, nebulizations will not be administered to avoid the inconvenience of sleep disruption. All participants will be on continuous oxygen saturation monitors.

### 2.5 Discontinuation criteria

Participants can withdraw from the study, if they wish, at any time and regardless of the reason, without consent of the research team. Any type of severe adverse events, according to the rules of the Common Terminology Criteria for Adverse Events (CTC-AE 5.0),^[30]^ will be considered criteria for discontinuation.

The reasons for discontinuing treatment will be properly documented by the research team and, in case the study is closed, the researchers will ensure the continuity of the medical and hospital assistance to the participant.

If COVID-19 is not confirmed by RT-PCR within 72 hours of the inclusion, the patient will be withdrawn from the study.

### 2.6 Outcomes: primary objectives assessment

1. Safety: related to the use of nebulized enriched heparin by patients with SARS-CoV-2 by the assessment of hemorrhagic events of any nature, alteration of the coagulation testing, such as alteration of the coagulation testing as reflected in a prolonged aPTT, indicated by an increase in aPTT >1.5, and heparin-induced thrombocytopenia;
2. Efficacy: related to the proposed treatment, by the analysis of the SARS-CoV-2 viral load of the participants treated for 7 days, by the sequential assessment of RT-PCR in the nasal swab, that will be compared between pre-treatment, 2, 5, and 7 days (study termination).

### 2.7 Outcomes: secondary objectives assessment

1. Safety measured by respiratory, clinical, laboratorial, and tomographic findings as follows, in a number of patients, and hospitalization days (including hospital discharge day):
2. Supplemental oxygen therapy needed;
3. Mechanical pulmonary ventilation needed;
4. Renal failure development;
5. Development of major cardiovascular events such as pulmonary embolism and acute myocardial infarction;
6. Demand for ICU treatment;
7. Secondary pulmonary bacterial infections (bacterial pneumonia);
8. Deep vein thrombosis (DVT) by Doppler ultrasonography assessment;
9. Pancreatitis characterized by increase in amylase >200 U/L;
10. Demand for corticosteroid therapy such as hydrocortisone, dexamethasone, and other corticosteroids duo to inflammatory pulmonary disease;
11. Death;
12. Increase in white blood cell count (>10,000 cells/mm^3^);
13. Increase in C reactive protein test (CRP) (>3.00 mg/L);
14. Deterioration of arterial blood gas paO_2_/pFiO_2_ ratio (<200);
15. Altered sodium (< 135 or > 145 mEq/L);
16. Altered potassium (< 3.5 or > 5.5 mEq/L);
17. Worsening of the pulmonary area compromised (%) by inflammation or infection by tomographic assessment.

### 2.8 Participant timeline

The closure assessment will be carried out on day 8. Randomization will be performed in up to 48 hours after admission. After the end of the study, the participant will be referred and accompanied at the COVID-19 outpatient clinic São Paulo State University (UNESP) Clinical Hospital (Table 1).

**Table 1:** Schedule for assessments and intervention.

Activities	Day 0	Day 1	Day 2	Day 3	Day 4	Day 5	Day 6	Day 7	Day 8
Informed consent form	X								
Medical visit, patient story and physical examination	X	X	X	X	X	X	X	X	X
Vital signs assessment	X	X	X	X	X	X	X	X	X
Medications in use	X	X	X	X	X	X	X	X	X
Inclusion and exclusion criteria	X								
COVID-19 viral load in RT-qPCR	X		X			X		X	X
Complete blood count, D-dimer, aPTT, urea, creatinine, glucose, reactive protein C, blood gas, and amylase	X		X			X		X	X
Computed thoracic tomography	X								X
HMWH or placebo administration		X	X	X	X	X	X	X	
Adverse events	X	X	X	X	X	X	X	X	X
Discontinuation criteria	X	X	X	X	X	X	X	X	X

The participants will be followed up and evaluated on days 0, 1, 2, 3, 4, 5, 6, 7, and 8 days after randomization.

### 2.9 Sample size and recruitment

The total recruitment size will be 50 participants, aiming at a minimum of 40 patients followed during the study period. The sample was estimated considering the number of patients diagnosed as COVID-19 at the São Paulo State University (UNESP) Clinical Hospital that met the study's eligibility criteria.

The minimum sample calculation of 40 patients took into account 2 independent study arms, with an estimated proportion in the viral load reduction rate of 30% for group 1, which will receive placebo, and 75% for group 2 that will be treated with enriched nebulized heparin as proposed in this study, considering 80% test power and a 5% significance level for a bilateral test.

### 2.10 Allocation

Participants will be divided into 2 groups and randomized 1:1. In this way, they will receive a sequential inclusion number from an electronically drawn number table and will receive the treatment according to the allocation in one of the groups:

The randomization list will be prepared by an independent statistician, using Stat Trek, a program to construct random number tables, available at http://stattrek.com/Tables/Random.aspx. Fifty positions will be drawn in a random and consecutive manner. The envelope containing the randomization will be kept in possession of an independent professional (pharmacist), so that the members of the study team will not know the order of allocation.

### 2.11 Blinding

The clinical trial will be triple-blind. Participants, researchers, the data, and statistical analysis team will not have access to the allocation numbers. A pharmacist will produce the heparin and the placebo and will assign a researcher of the team to distribute the product to each participant, according to the randomization list. In addition, presentation of the preparation to the control and experimental group will be identical, as enriched heparin is colorless and odorless to the human eye and nose, as well as the saline. The blinding code will not be broken until all research participants have completed the study and the database is closed, that is, it will not undergo further changes (Table 2).

**Table 2:** Common Terminology Criteria for Adverse Events (CTC-AE) 5.0 classification^[24]^.

Grade	Description
Grade 1: Mild	Asymptomatic or mild symptoms; clinical or diagnostic observations only; intervention not indicated.
Grade 2: Moderate	Minimal, local or noninvasive intervention indicated; limiting age-appropriate instrumental activities of daily life.
Grade 3: Severe or medically significant but not immediately life-threatening	Medical hospitalization or prolongation of hospitalization indicated; disabling; limiting self-care activities of daily life.
Grade 4: Life-threatening consequences	Urgent intervention indicated.
Grade 5: Death	Death related to adverse effects.

### 2.12 Emergency unmasking

In an emergency, and if necessary, members of the data and security monitoring committee can request a blinding break, only for the participant at risk and after evaluation of the security committee.

### 2.13 Data collection plan

Data will be collected daily by the researchers by filling of assessment forms.

All assessment forms are available as supplementary materials, http://links.lww.com/MD/G541: Epidemiological and personal antecedents form; Clinical assessment and physical examination form; Laboratory results and tomographic findings form; Adverse events form. (Supplement Material 1 - Figure 1, http://links.lww.com/MD/G537, Supplement Material 2 - Figure 2, http://links.lww.com/MD/G538, Supplement Material 3 - Figure 3, http://links.lww.com/MD/G539, Supplement Material 4 - Figure 4, http://links.lww.com/MD/G540).

### 2.14 Retention

The closure assessment will be carried out on day 8. Randomization will be performed in up to 48 hours after admission. After the end of the study, the participant will be referred and accompanied at the COVID-19 outpatient clinic São Paulo State University (UNESP) Clinical Hospital.

### 2.15 Statistics

Categorical variables will be evaluated with Fisher's exact test, continuous variables will be compared with non-parametric methods such as Mann–Whitney test or *U* test, paired comparisons will be evaluated with the Wilcoxon test, multivariate analysis will be evaluated with regression Cox analysis and long-term clinical analyzes will be compiled on Kaplan–Meir curves.

Missing data and multiple imputations will cause discontinuation of the patient enrolment in order to avoid bias.

### 2.16 Adverse effects

Adverse effects related to the nebulized use of heparins will be tracked and analyzed according to the Common Terminology Criteria for Adverse Events (CTC-AE 5.0),^[30]^ and will be graded from 1 to 5, according to the severity of the event.

Adverse effects secondary to the use of heparins will cause the discontinuation of the study if graded 3 or more. All severe adverse effects noted by the researchers will be reported to the independent data and security monitoring committee.

The following data will be analyzed in case of an adverse event: the type of event or reaction, including predefined events (major bleeding, pulmonary bleeding, and heparin-induced thrombocytopenia); start date and time; date and time of the most recent administration of heparin; the extent of any causal link to the enriched heparin; the severity of the event.

The following adverse events and reactions will be considered severe: Any event that is related to the use of enriched heparin and causes prolongation of hospital stay, disabling, limiting self-care activities, urgent interventions needed and death; bleeding that results in death, that occurs in a critical area or organ (intracranial, spinal, intraocular, retroperitoneal, intra-articular, or intramuscular), which results in a drop in hemoglobin of 20 g/L or more or demands transfusion of 2 or more units of whole blood or red blood cells; lung bleeding, which is the notable bleeding from the lungs, trachea, or bronchi with repeated hemoptysis or which requires repeated aspiration and is associated with acute deterioration of the respiratory state; heparin-induced thrombocytopenia, which is an unexplained drop in platelet count and a positive heparin antibody test; any other adverse event and reaction that, after evaluated by the independent data and security monitoring committee, are considered not part of the expected clinical course and may be related to the study.

### 2.17 Patient and public involvement

This research was designed to evaluate the safety of a well stablished drug, heparin, for a novel purpose. This trial and its questions are in line with a moment of patient's high expectations for treatment options for COVID-19 pneumonia. Drug efficacy and low cost are of paramount importance, considering the extension and raw number of patients worldwide, particularly in developing countries.

To achieve a minimum level of bias, we designed a triple-blind study, in which patients are recruited after in-hospital admission and are fully aware of its methodology, risks, and benefits. All patients are oriented of heparin's primal role as an anticoagulant, although the proposed dosage and the nebulized administration has already shown not to increase thromboplastin time, and, therefore, does not induce anticoagulation.

The burdens of intervention will be assessed daily by clinical evaluations and laboratory tests, and patients can report any signals or symptoms at any given moment.

### 2.18 Strengths and limitations

This is the first clinical trial that uses inhaled enriched heparin, which allows a lower dosage of heparin to be administered in comparison to other ongoing trials.

It is a triple-blind randomized study.

Dropout rate may be high.

Hospitalization time may be affected, as laboratory exams will be assessed for 7 days.

### 2.19 Ethics and dissemination

The EnHanCed clinical trial will be conducted in accordance with all applicable Brazilian national laws and international guidelines; in accordance with the ethical principles defined in the 18th World Medical Assembly, Helsinki, 1964, and all applicable amendments; following the International Council for Harmonization (ICH) guidelines for Good Clinical Practice (GCP).

EnHanCed is registered on Brazilian and international platforms under the following registration IDs:

World Health Organization (WHO) International Clinical Trials Registry Platform (ICTRP) Universal Trial Number (UTN): U1111-1264-8253, submitted under the Brazilian Clinical Trials Registry (ReBEC).

National Institute of Health (NIH) United States National Library of Medicine ClinicalTrials.gov platform ID: NCT04743011

## Author contributions

**Conceptualization:** Matheus Bertanha, Andrei Moroz, Maria Inês de Moura Campos Pardini, Edison Luiz Durigon, Rafael Rahal Guaragna Machado, Marcelo Andrade de Lima, Helena Bonciani Nader, Marli Leite de Moraes, Angelo José Magro, Cristiane Rodrigues Guzzo Carvalho, Rita de Cássia Alvarado, Carlos Magno Castelo Branco Fortaleza.

**Data curation:** Lenize da Silva Rodrigues, Marcone Lima Sobreira, Alexandre Naime Barbosa, Natália Bronzatto Medolago, Helga Caputo Nunes, Gustavo Constantino de Campos.

**Formal analysis:** Helga Caputo Nunes, Gustavo Constantino de Campos.

**Funding acquisition:** Matheus Bertanha.

**Investigation:** Matheus Bertanha, Pedro Luciano Mellucci Filho, Vinicius Tadeu Ramos da Silva Grillo, Nathalia Dias Sertorio.

**Methodology:** Matheus Bertanha, Pedro Luciano Mellucci Filho, Andrei Moroz, Maria Inês de Moura Campos Pardini, Edison Luiz Durigon, Rafael Rahal Guaragna Machado, Rejane Maria Tommasini Grotto, Marcelo Andrade de Lima, Helena Bonciani Nader, Marli Leite de Moraes, Natália Bronzatto Medolago, Angelo José Magro, Cristiane Rodrigues Guzzo Carvalho, Rita de Cássia Alvarado, Carlos Magno Castelo Branco Fortaleza.

**Project administration:** Matheus Bertanha, Carlos Magno Castelo Branco Fortaleza.

**Resources:** Lenize da Silva Rodrigues, Alexandre Naime Barbosa, Fábio Florença Cardoso, Leonardo Nazário de Moraes.

**Software:** Pedro Luciano Mellucci Filho.

**Supervision:** Lenize da Silva Rodrigues, Marcone Lima Sobreira, Marcelo Andrade de Lima, Alexandre Naime Barbosa, Carlos Magno Castelo Branco Fortaleza.

**Validation:** Andrei Moroz, Maria Inês de Moura Campos Pardini, Edison Luiz Durigon, Rafael Rahal Guaragna Machado, Rejane Maria Tommasini Grotto, Helena Bonciani Nader, Marli Leite de Moraes, Fábio Florença Cardoso, Angelo José Magro, Cristiane Rodrigues Guzzo Carvalho, Leonardo Nazário de Moraes, Rita de Cássia Alvarado, Carlos Magno Castelo Branco Fortaleza.

**Visualization:** Marcelo Andrade de Lima.

**Writing – original draft:** Matheus Bertanha, Pedro Luciano Mellucci Filho.

**Writing – review & editing:** Matheus Bertanha, Marcelo Andrade de Lima, Natália Bronzatto Medolago, Carlos Magno Castelo Branco Fortaleza.
